# Tensile and Tearing Properties of a Geocomposite Mechanically Damaged by Repeated Loading and Abrasion

**DOI:** 10.3390/ma16217047

**Published:** 2023-11-05

**Authors:** José Ricardo Carneiro, Filipe Almeida, Filipa Carvalho, Maria de Lurdes Lopes

**Affiliations:** CONSTRUCT, Department of Civil Engineering, Faculty of Engineering, University of Porto, Rua Dr. Roberto Frias, 4200-465 Porto, Portugal; filipe.almeida@fe.up.pt (F.A.); ec09004@fe.up.pt (F.C.); lcosta@fe.up.pt (M.d.L.L.)

**Keywords:** geosynthetics, geocomposites, degradation, mechanical damage under repeated loading, abrasion, tensile behaviour, tearing behaviour, reduction factors

## Abstract

The behaviour of geosynthetics can be affected by many agents, both in the short and long term. Mechanical damage caused by repeated loading or abrasion are examples of agents that may induce undesirable changes in the properties of geosynthetics. The research conducted in this work complemented previous studies and consisted of submitting a geocomposite, isolated and successively, to two degradation tests: mechanical damage under repeated loading and abrasion. The geocomposite (a nonwoven geotextile reinforced with polyethylene terephthalate filaments) was tested on both sides (with or without filaments) and directions (machine and cross-machine). The impact of the degradation tests on the geocomposite was quantified by monitoring changes in its tensile and tearing behaviour. The results showed that, in most cases, the degradation tests caused the deterioration of the tensile and tearing behaviour of the geocomposite, affecting its reinforcement function. The decline in tensile strength correlated reasonably well with the decline in tearing strength. Changing the side and direction tested influenced, in some cases (those involving abrasion), the degradation experienced by the geocomposite. The reduction factors (referring to tensile and tearing strength) for the combined effect of the degradation agents tended to be lower when determined by using the common method (compared to those resulting directly from the successive exposure to both agents).

## 1. Introduction

Reinforcing soils with geosynthetics is a common practice in many civil engineering works. Geosynthetics are applied as reinforcements to improve the mechanical properties of soils and prevent inadmissible deformations in geotechnical structures [1]. The reinforcing function can be performed by using different geosynthetics, typically geogrids, geotextiles or geocomposites [2]. To carry out this function, geosynthetics not only need to have adequate mechanical properties (high strength and stiffness) but also maintain minimum values of these properties for a usually long period of time.

Like other construction materials, geosynthetics can degrade over time. Their degradation agents, which can vary from application to application, are various, including installation damage, weathering, oxidation, heat, hydrolysis, abrasion or creep [3]. The action of degradation agents, which can affect the performance of geosynthetics over time, must be considered during the design phase of the applications involving the use of these materials. ISO/TR 20432 [4] provides guidance for determining the long-term strength of geosynthetics for soil reinforcement. This technical report specifies a method for deriving reduction factors that account for the effects of installation damage, creep and creep rupture, weathering and chemical and biological degradation. Partial reduction factors (one for each agent or type of degradation) are obtained in isolation, with the overall effect of the degradation agents being obtained by multiplying the various partial reduction factors. Similar procedures for calculating reduction factors can be found in other documents [5,6,7]. Previous research has shown that, although it is a common method, multiplying partial reduction factors may result in a misrepresentation of the combined effect of degradation agents. When inaccurate, the resulting reduction factors may either be conservative [8,9,10] or underestimate [11,12,13] the combined effect of the degradation agents.

Even if done carefully and following best practices, the installation of geosynthetics, as well as the placement and compaction of soils over them, may cause mechanical damage (e.g., cuts or holes) to these construction materials. In many cases, geosynthetics can experience the highest mechanical stresses during installation [1,14]. Installation activities can induce abrasion in geosynthetics, e.g., by the use of vibratory soil compactors, triggering the mobilization of frictional forces between these materials and soil. However, abrasion can actually be a permanent degradation agent in some applications. This is the case of roadways and railways infrastructures, where daily traffic of vehicles can materialize a cyclic load over time that may cause abrasion in geosynthetics.

The resistance of geosynthetics to installation activities can be evaluated by using field tests (e.g., [14,15,16,17]) or laboratory tests (e.g., [18,19,20,21]). Field tests normally involve installation under real conditions, providing reliable results on the survivability of geosynthetics. However, they are often expensive and time-consuming and require heavy equipment. Alternatively, laboratory tests can be used to assess the resistance of geosynthetics to mechanical damage. These tests, of which the one described in EN ISO 10722 [22] is an example, are more expeditious but less specific and provide less accurate results than the field tests under real installation conditions. There are also laboratory methods to determine the resistance of geosynthetics to abrasion, the method described in EN ISO 13427 [23] (sliding block test) being an example. The damage experienced by the geosynthetics (in the field or laboratory tests) is typically quantified by monitoring changes in their properties, often tensile (e.g., [14,15,16,17,18,19]) and/or hydraulic (e.g., [20,21]) properties.

Previous research on mechanical damage of geosynthetics promoted by exposure to repeated loading and abrasion has shown that the mechanical and hydraulic properties of these materials can be affected by damaging actions [12,13,24]. Different materials have been tested, mostly geotextiles and geogrids, with their structure having a significant influence on their survivability to damaging actions. Although extrapolating laboratory results to reality is complicated, these results can be an indicator of the resistance of geosynthetics to degradation, allowing the behaviour of different materials to be compared.

This work follows a previous study [24] on the resistance of a reinforcement geocomposite to mechanical damage induced by repeated loading and abrasion, complementing it and including a significant amount of additional results. As will be described later, the geocomposite was formed by two different elements, resulting in distinct structures on each side (the structure also varied with direction). The geocomposite was tested on both sides and directions, with the effect of the degradation tests being measured by their impact on its tensile and tearing behaviour. The main goals of the work included: (1) assess how changing the side and direction tested influenced the degradation suffered by the geocomposite; (2) evaluate how the different elements of the geocomposite were affected by the damaging actions; (3) find out whether changes in the tensile and tearing strength of the geocomposite could be related; and (4) compare methods for determining reduction factors for the combined effect of the degradation agents.

## 2. Materials and Methods

### 2.1. Testing Material

**Figure 1:**
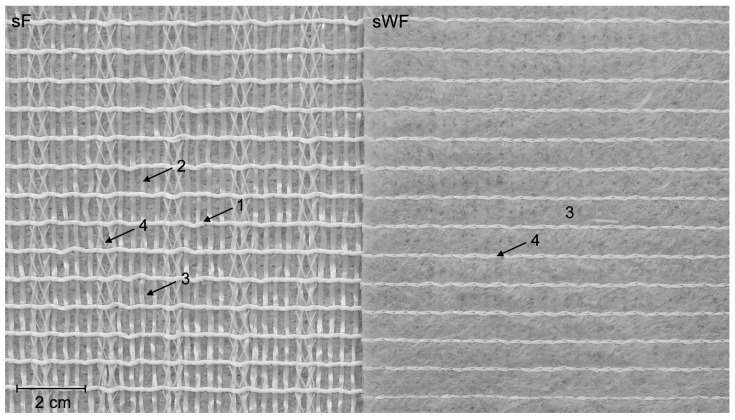
Geocomposite (intact sample): side with filaments and side without filaments. 1—MD filament; 2—CMD filament; 3—geotextile; 4—seam.

The geocomposite was formed by a polypropylene (PP) nonwoven geotextile reinforced with polyethylene terephthalate (PET) filaments. As illustrated in Figure 1, the filaments were fixed by seams to the geotextile and, although with a different arrangement, were present both in the machine direction (MD) and cross-machine direction (CMD) of production. The sets of filaments in the MD were thicker but further apart (in less quantity) than those in the CMD. Despite the different arrangement of filaments, the geocomposite had a tensile strength of 42 kN·m^−1^ (value reported by the manufacturer) in both directions. Figure 1 also shows that the geocomposite had sides with different characteristics: one with filaments and the other without filaments (side with the geotextile). For ease of writing, the different sides of the geocomposite are going to be designated, respectively, by sF (side with filaments) and sWF (side without filaments). Also for simplicity, the filaments arranged in the MD will be called MD filaments, with those arranged in the CMD being designated as CMD filaments.

Before the degradation tests, the geocomposite (intact sample) was characterised. Its tensile and tearing properties can be found in Section 3.2 and Section 3.3. Other properties of the geocomposite were as follows (values presented with 95% confidence intervals): mass per unit area: 351 ± 16 g·m^−2^; thickness at 2 kPa: 2.26 ± 0.09 mm; permittivity (head loss of 50 mm): 74.4 ± 8.6 mm·s^−1^; pore size (*O*_90_): 94 ± 6 µm. The previous properties were obtained in accordance with, respectively, EN ISO 9864 [25], EN ISO 9863-1 [26], EN ISO 11058 [27] and EN ISO 12956 [28]. In all cases, the procedures for sampling and preparation of test specimens were carried out according to EN ISO 9862 [29]. This standard was also followed when preparing specimens for degradation tests (Section 2.3).

Durability aspects of the geocomposite were also evaluated. The selected tests followed the indications of EN 13249 [30] and EN 13250 [31], standards that specify the characteristics of geosynthetics for their use in the construction of, respectively, roads and railways—two applications where they may experience abrasive actions over time. Results showed that the geocomposite had good resistance to acids and alkalis (EN ISO 12960 [32]), hydrolysis (EN 12447 [33]) and oxidation (EN ISO 13438 [34])—in all tests, retained strength close to 100%. Results on the resistance of the geocomposite to weathering can be found in Carneiro et al. [35].

### 2.2. Work Plan

The geocomposite was submitted to the following degradation conditions: (1) mechanical damage under repeated loading (sample RL); (2) abrasion (sample A); (3) double testing condition (sample RL-A), which consisted of mechanical damage under repeated loading followed by abrasion.

As the geocomposite had sides with different characteristics (as illustrated in Figure 1), both sides (sF and swF) were tested. The work plan also considered the effect of testing the geocomposite in different directions: MD and CMD. Thus, to identify the tested direction and side, four designations were used: MD-sF, MD-sWF, CMD-sF and CMD-sWF.

The work involved testing 200 geocomposite specimens according to the plan illustrated in Table 1. Of these 200, 150 were submitted to degradation tests previously to their characterisation by tensile or tearing tests (samples RL, A and RL-A). The other 50 (intact sample) were characterised immediately (also by tensile or tearing tests), with their properties functioning as a reference to evaluate the damage caused by the degradation tests. Of the 150 specimens submitted to degradation tests, 60 were tested in the MD (for subsequent characterisation by tearing tests) and 90 in the CMD (30 for subsequent characterisation by tensile tests and the other 60 by tearing tests). The tensile behaviour of specimens tested in the MD was reported in Carneiro et al. [24] and hence the smaller number of specimens tested in the MD in this work (compared to those tested in the CMD). The degradation and characterisation tests were carried out under the temperature and relative humidity conditions recommended in the respective test standards.

### 2.3. Degradation Tests

The mechanical damage under repeated loading tests (for ease of writing, hereinafter designated as RL tests) and the abrasion tests followed standard methods, which, in addition to being described in the respective standards (EN ISO 10722 [22] and EN ISO 13427 [23], respectively), were also described in previous works [12,13,24]. Therefore, in this work, only a short description of these tests is provided.

The RL tests consisted of submitting the geocomposite, which was installed between two layers of *corundum* (synthetic aggregate from aluminium oxide), to dynamic loading between (5.0 ± 0.5) kPa and (500 ± 10) kPa at a frequency of 1 Hz, for 200 cycles. *Corundum* had a particle size distribution ranging from 5 to 10 mm. As mentioned previously, the geocomposite was tested on both sides. In the RL tests, the tested side was defined as the side of the geocomposite facing the load application mechanism.

In abrasion tests, the geocomposite was rubbed with a P100 abrasive. The abrasion process was conducted under a pressure of 6 kPa and included 750 abrasion cycles. A schematic of the abrasion tests can be found in Carlos et al. [13]. In these tests, the tested side refers to the side of the geocomposite in direct contact with the P100 abrasive.

### 2.4. Tensile and Tearing Tests

The damage suffered by the geocomposite in the degradation tests was assessed by monitoring changes in its tensile and tearing behaviour. Tensile tests (conducted only in the CMD) and tearing tests (conducted both in the MD and CMD) were performed on the geocomposite according to, respectively, EN ISO 10319 [36] and ASTM D4533 [37]. These tests were carried out under displacement rates of, respectively, 20 and 300 mm·min^−1^.

The specimens used in the tensile tests had a length of 100 mm (between grips) and a width of 200 mm. Regarding the tearing tests, the specimens were 76 mm wide and 200 mm long, with an isosceles trapezoid (little base of 25 mm and big base of 100 mm) marked on their centre. Before testing, a 15 mm tear was made in the middle of the little base (the test measures the force required to propagate this tear). The gripping area corresponded to the area outside the trapezoid.

Tensile strength (maximum tensile force per unit width) (*T*, in kN·m^−1^) and elongation at tensile strength (*Ɛ_T_*, in %) were the properties determined in the tensile tests. Elongation was measured by using a video extensometer, which followed the movement of two reference points installed 30 mm above and 30 mm below the centre of symmetry of the specimens. The tearing tests allowed the tearing strength (*F_T_*, in N) to be obtained. The values of the properties resulting from the tensile and tearing tests correspond to the arithmetic means of, respectively, 5 and 10 tested specimens and are presented with 95% confidence intervals. In some cases, tensile and tearing strength results are presented as percentage residual values, obtained by dividing the strength of the damaged samples by that of the intact sample. The tensile and tearing strength results also allowed the calculation of reduction factors (RFs) for the effect (isolated and combined) of the RL and abrasion tests. As in other works [12,13,24], the reduction factors represent the quotient between the strength (tensile or tearing) of the intact sample and the respective strength of a damaged sample.

## 3. Results and Discussion

### 3.1. Visual Analysis

**Figure 2:**
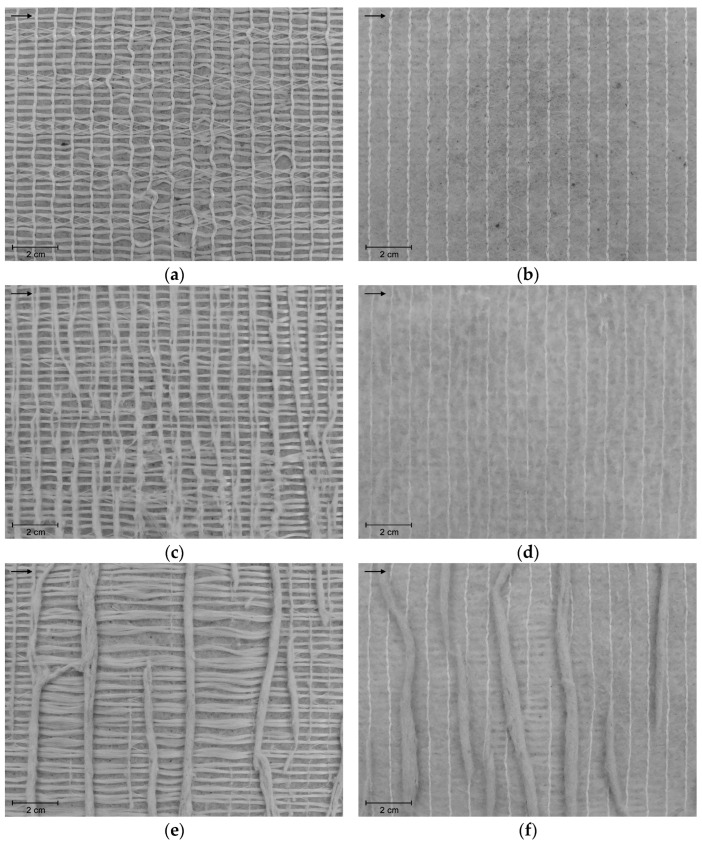
Damaged geocomposite samples (tests in the CMD): (**a**) sample RL: sF; (**b**) sample RL: sWF; (**c**) sample A: sF; (**d**) sample A: sWF; (**e**) sample RL-A: sF; (**f**) sample RL-A: sWF. Note: the arrow indicates the CMD.

The geocomposite underwent changes during the degradation tests, which were different from test to test. In most cases, damage detected visually (with the naked eye) immediately indicated that the mechanical behaviour of the geocomposite had deteriorated, with its reinforcement function clearly affected. Table 2 provides a qualitative summary of the damage found in the geocomposite after the degradation tests, while Figure 2 shows examples of damaged geocomposite samples (both Table 2 and Figure 2 include results obtained for samples tested in the CMD). Results for samples tested in the other direction can be found in Carneiro et al. [24].

### 3.2. Tensile Behaviour

**Figure 3:**
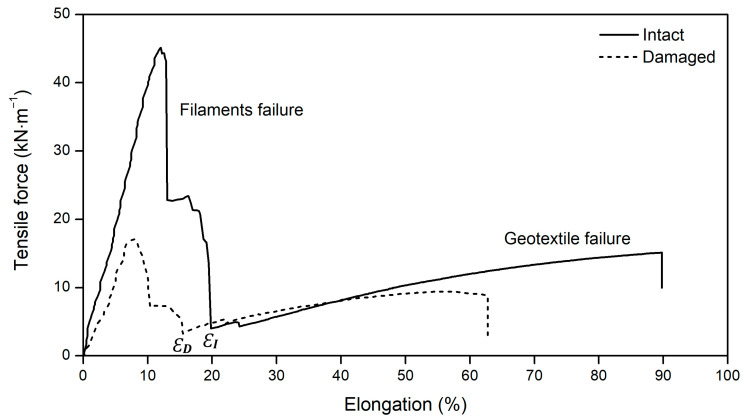
Tensile force–elongation curves of the geocomposite (examples in the CMD).

The geocomposite was formed by two elements with different tensile behaviour: the PET filaments and the PP geotextile. The filaments had greater resistance but were significantly less deformable than the geotextile. When performing tensile tests, the difference between the tensile behaviour of the filaments and geotextile resulted in two distinct areas in the force–elongation curve of the geocomposite. Figure 3 exemplifies two tensile force–elongation curves of the geocomposite, one referring to an intact sample and the other to a damaged sample. Both curves were obtained for samples tested in the CMD.

As illustrated in Figure 3, the main contribution to the mechanical resistance of the geocomposite was provided by the filaments (reinforcing elements). Their failure (under tensile load) occurred at low elongation. After that, the tensile test continued on the highly deformable (but less resistant) geotextile, which failed at relatively high elongation. The symbols *Ɛ_I_* and *Ɛ_D_* in Figure 3 denote, respectively, for the intact and damaged samples exemplified, the moment from which the tensile test continued only on the geotextile (the filaments were all broken, not contributing to the tensile force from then on). The results of the tensile tests performed on the geocomposite (intact and damaged samples) are provided in Table 3 (tensile tests carried out in the CMD).

The results presented in Table 3 show that the degradation tests induced changes in the tensile behaviour of the geocomposite. As can be seen from the low elongation values, the tensile strength was, in all cases, reached at the time of filament failure (initial peak in the tensile force–elongation curves represented in Figure 3). The next subsections will discuss in detail the tensile results obtained for the geocomposite in the CMD. It should be remembered that the results obtained in the MD can be found in Carneiro et al. [24].

#### 3.2.1. Effect of Exposure Side on PET Filament Failure

- Tests on the Side with Filaments

When tested on the sF, the tensile behaviour of the geocomposite was affected differently by the degradation tests (Table 3). The tensile strength of the RL sample was considerably lower than that of the intact sample (reduction of 58.5%). This outcome can be explained by the damage induced by the rough and angular particles of *corundum*, which provoked cuts in some CMD filaments, affecting meaningfully the tensile strength of the geocomposite.

The reduction in tensile strength experienced by the A sample (9.3%) was considerably lower than that suffered by the RL sample. As illustrated in Figure 1, the MD filaments were arranged above the CMD filaments, preventing the latter from being in direct contact with the P100 abrasive. Therefore, the MD filaments protected the CMD filaments (mainly responsible for the mechanical resistance of the geocomposite in the CMD) from suffering extensive degradation.

The RL-A sample suffered the most pronounced reduction in tensile strength, namely 72.8%. Considering the visually detected damage (Figure 2 and Table 2), which was more severe than in samples RL and A, the greater impact of the double exposure condition on the tensile behaviour of the geocomposite was expected.

Regarding the elongation at tensile strength, despite the 95% confidence intervals being relatively wide, this parameter tended to be smaller in the damaged samples (i.e., RL, A and RL-A samples) than in the intact sample.

- Tests on the Side without Filaments

When tested on the sWF, the tensile properties of the geocomposite were also affected by the degradation tests (Table 3). The tensile strength of the RL sample was 40.9% lower than that of the intact sample. This reduction was less pronounced than that found when the geocomposite was tested on the sF (reduction of 58.5%). Considering that (1) regardless of the side tested (side directed towards the load application mechanism), there was *corundum* on both sides of the geocomposite and (2) visually, the damage induced to the geocomposite was similar regardless of the side tested, it is difficult to explain the above difference in the reduction in tensile strength. It should be noted that, when tested in the MD, changing the test side had no meaningful influence on the degradation suffered by the geocomposite [24]. Furthermore, as will be seen ahead, both for the tensile behaviour of the geotextile (following the failure of the filaments) and for the tearing strength of the geocomposite, there was no marked influence of changing the side of the geocomposite directed towards the load application mechanism.

Advancing to sample A, the abrasive actions caused a reduction in tensile strength of only 4.1%, a value slightly lower (although not significantly different considering the 95% confidence intervals) than that found when the geocomposite was tested on the sF. The CMD filaments were once again protected from degradation, but now by the geotextile, which was the element in direct contact with the P100 abrasive.

As on the sF, the double testing condition caused the most pronounced reduction in the tensile strength of the geocomposite, namely 62.5%. This reduction was smaller than that found when the geocomposite was tested on the sF (72.8%), which is in line with the also smaller reductions observed when comparing the RL and A samples.

The elongation at tensile strength of the damaged samples was very close (between 7.8% and 8.3%) and tended to be lower than that of the intact sample. However, the 95% confidence intervals were again relatively wide.

#### 3.2.2. PP Geotextile Failure vs. PET Filament Failure

As previously mentioned, after the failure of the filaments (which broke at low elongations: below 10%), the tensile test continued on the geotextile. In all cases (intact and damaged samples), the maximum tensile force (tensile strength) was reached at the time of filament failure. Therefore, the tensile results presented in the previous subsection do not provide indications about the effect of the degradation conditions on the geotextile, whose failure occurred at high elongations (above 80% in the intact sample). The values of the maximum tensile force (*F*), and respective elongation (*Ɛ_F_*), of the geotextile (corresponding to the last peak in the tensile force–elongation curves represented in Figure 3) can be found in Table 4 (values obtained in the CMD).

Table 4 shows that the tensile behaviour of the geotextile was affected by the degradation tests. In the RL and RL-A samples (tested on the sF), the maximum tensile force of the geotextile was relatively close, however, significantly lower than that of the intact sample: reductions of 41.6% and 46.2%, respectively. The corresponding elongations were also considerably lower than that of the intact sample. The damage caused by the angular and rough particles of *corundum* (namely, cuts in fibres, punctures and holes) explains the deterioration of the tensile behaviour of the geotextile. Regarding sample A (also tested on the sF), there was no pronounced decrease in the maximum tensile force of the geotextile (reduction of 7.2%), proving that this component was not greatly affected by abrasion, as indicated by visual inspection. A relatively minor reduction was also found in the elongation at which the maximum tensile force was registered. The relatively small effect of abrasion can be explained by the fact that, when the geocomposite was tested on the sF, the geotextile did not come into direct contact with the P100 abrasive (there were filaments on top of the geotextile). This low effect of abrasion on the geotextile can also be observed when comparing the results obtained for samples RL and RL-A, where exposure to abrasion of the sample previously submitted to RL tests did not result in very significant additional damage.

When the geocomposite was tested on the sWF, the tensile behaviour of the geotextile also underwent relevant changes, although different from those observed on the sF. In the RL sample, the reduction (34.8%) in the maximum tensile force of the geotextile was not very different from that observed when the geocomposite was tested on the sF (41.6%). Indeed, taking into account the 95% confidence intervals, the tensile force values were not significantly different. The reasons for the decrease in the maximum tensile force of the geotextile are the same as those presented previously when the geocomposite was tested on the sF (regardless of the side directed to the load application mechanism, both sides of the geocomposite were in contact with *corundum*). The effect of abrasion on the geotextile was slightly more noticeable when the geocomposite was tested on the sWF. Indeed, a higher reduction (namely 16.5%) occurred in the maximum tensile force of the geotextile (compared to a 7.2% reduction when the geocomposite was tested on the sF). This more pronounced impact can be explained by the fact that, in this case, the geotextile was in direct contact with the P100 abrasive and therefore more prone to be degraded. Still, the greatest effect of changing the test side of the geocomposite was observed in the RL-A sample. In this case, the geotextile previously damaged by *corundum* was considerably affected by abrasion. The double exposure condition led to a 76.9% reduction in the maximum tensile force of the geotextile.

**Figure 4:**
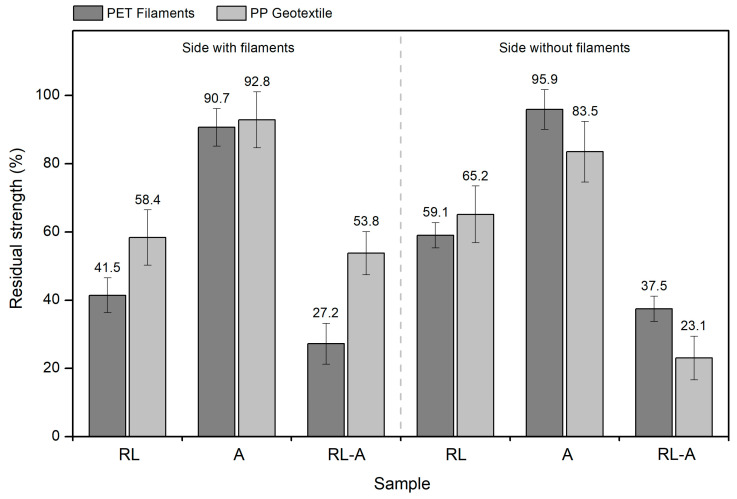
Tensile strength of the geocomposite (PET filaments) vs. maximum tensile force of the PP geotextile (percentage residual values). Note: results obtained in the CMD.

Having analysed the effect of the degradation conditions on the tensile behaviour of the geotextile, it is relevant to compare it with what happened to the filaments. Thus, Figure 4 compares, in percentage residual values, the tensile strength of the geocomposite (for which the filaments were the main responsible) with the maximum tensile force of the geotextile.

As can be seen in Figure 4, although there are some differences, the effect of the degradation conditions on the strength of the filaments and geotextile was relatively close (in terms of percentage changes). It should be remembered that the results presented in Figure 4 correspond to the CMD, and, therefore, the filaments under analysis (responsible for the tensile strength of the geocomposite) are the CMD filaments. In sample RL, the reduction in tensile force was more marked in the filaments than in the geotextile, showing that the latter withstood better the damaging actions. Regarding sample A, both the geotextile and filaments were not extremely impacted, and therefore, no pronounced reductions were found in tensile force. Still, the highest difference between the residual strength of the filaments and the geotextile was observed when the geocomposite was tested on the sWF, the case where the geotextile was in direct contact with the P100 abrasive and, therefore, was slightly more affected. Finally, in sample RL-A, the filaments underwent higher degradation (higher reduction in tensile force) when the geocomposite was tested on the sF, with the opposite occurring when the tests were carried out on the sWF. In the latter case, the geotextile (already damaged by the RL tests) was directly exposed to the abrasive actions.

#### 3.2.3. Cross-Machine Direction vs. Machine Direction

**Figure 5:**
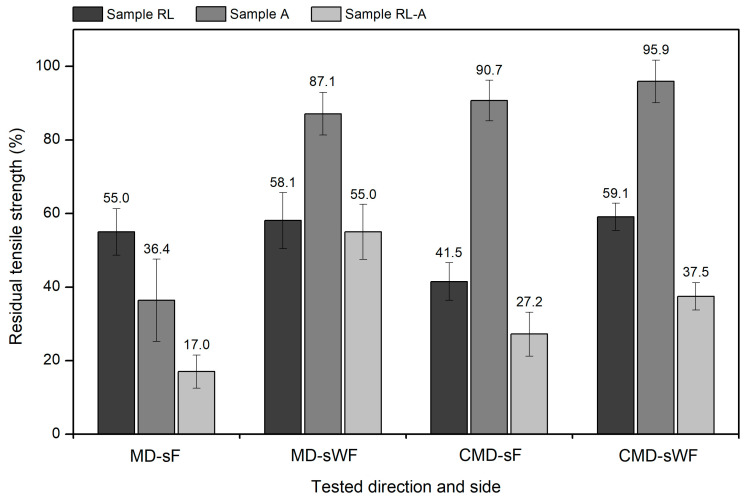
Residual tensile strength of the geocomposite: MD vs. CMD.

As presented in the previous sections, the tensile behaviour of the geocomposite was, in most cases, considerably affected by the degradation conditions. Figure 5 compares the tensile results (residual tensile strengths) obtained in this work (geocomposite tested in the CMD) with those found in the MD (reported in Carneiro et al. [24]).

In tests carried out on the sF, the residual tensile strength of the RL sample was higher in the MD (55.0%) than in the CMD (41.5%). This difference may be related to the arrangement of the filaments, which differed in the MD and CMD. As shown in Figure 1, in the MD, there were relatively thick strands, apparently with higher resistance to damage. By contrast, in the CMD, the filaments were untied and arranged in smaller sets, therefore more likely to be affected. Interestingly, this behaviour was not replicated when the geocomposite was tested on the sWF (i.e., with the geotextile directly facing the load application mechanism). In this last case, the residual tensile strengths were 58.1% (MD-sWF) and 59.1% (CMD-sWF).

Returning to testing on the sF, the tensile behaviour of sample A differed significantly depending on the direction tested. Indeed, as illustrated in Figure 5, the residual tensile strength was approximately 2.5 times lower in the MD than in the CMD (36.4% and 90.7%, respectively). This outcome is related to the MD filaments being placed above the CMD filaments, preventing the latter from being in direct contact with the P100 abrasive (and thus protecting them from suffering extensive damage). This difference was not observed when the geocomposite was tested on the sWF. In the latter case, regardless of the direction tested, it was the geotextile that was in direct contact with the P100 abrasive. Consequently, the residual tensile strength of sample A was very close when the geocomposite was tested in the MD (87.1%) and CMD (95.9%). The results obtained for sample A show that, for geocomposites (or other materials with different sides), abrasion resistance must be tested on both sides, with the possibility of obtaining different results—the standard abrasion test [23] only allows damage to be induced on one side at a time. For geosynthetics with different structures in the MD and CMD (such as the geocomposite under study), it is also important to evaluate the abrasion resistance in both directions.

Finally, there is the case of the RL-A sample to analyse. When the geocomposite was tested on the sF, as in sample A (although less pronounced), its residual tensile strength was higher in the CMD (27.2%) than in the MD (17.0%). This behaviour can be explained by the degradation of MD filaments during the abrasion tests being more severe than that of CMD filaments. By contrast, when tests were performed on the sWF, the previous behaviour was reversed, i.e., a more marked decrease in tensile strength (sample RL-A) was observed when the geocomposite was tested on the CMD—residual tensile strengths of 55.0% (MD-sWF) and 37.5% (CMD-sWF).

Overall, geosynthetics that have different characteristics on both directions and sides (an aspect that is related to their manufacturing process and resulting structure) can suffer distinct degradation (measured here by changes in tensile strength) depending on the direction and side tested. In the geocomposite under study, and for the reasons already discussed, this was mostly noticed when the degradation conditions involved exposure to abrasion.

### 3.3. Tearing Behaviour

The degradation tests conducted on the geocomposite also promoted changes in its tearing behaviour. The results presented in Table 5 show that the different testing conditions led to different outcomes depending on the direction and side of the geocomposite tested. It should be noted that, in some cases, the tearing strength values (Table 5) had a relatively high dispersion, as demonstrated by the width of the 95% confidence intervals. The results of the tearing tests are going to be analysed in the following two subsections.

#### 3.3.1. Tests in the Machine Direction

When tested on the sF, the RL sample suffered a decrease in tearing strength of 48.8%. This outcome can be explained by the existence of cuts in some filaments, as well as cuts in fibres, punctures and holes in the geotextile (damage induced by the rough and angular particles of *corundum*). The decrease in tearing strength observed in sample A (43.9%) was very close to that found in sample RL, however, for different reasons. In this case, the deterioration in tearing behaviour can be allocated to the P100 abrasive, which, among other things, promoted the detachment and cut of MD filaments. The 75.2% reduction in tearing strength found in the RL-A sample was more substantial than the reduction that occurred in the RL and A samples. Considering the previous results, this was an expected outcome since the double testing condition consisted in submitting a sample already weakened by the RL tests to abrasive actions, which left the geocomposite deeply damaged.

The results obtained when the geocomposite was tested on the sWF had some differences compared to those found when the tests were performed on the sF, essentially due to the different degradation induced by the P100 abrasive. Accordingly, regarding the RL sample, there were no significant differences in the reduction in tearing strength when the tests were carried out on the sF or sWF (reductions of, respectively, 48.8% and 46.1%). This result showed that changing the side of the geocomposite directly facing the load application mechanism had no marked influence on the deterioration of its tearing behaviour. The same outcome was obtained for its tensile behaviour [24].

As mentioned, the change in the tested side influenced the degradation caused by the abrasion tests: samples A and RL-A. In sample A (sWF), the filaments were apparently unharmed by the abrasive actions (a feature resulting from the direct exposure of the geotextile to the P100 abrasive), which resulted in only a small reduction in tearing strength (8.6%)—a very different reduction from that observed when the geocomposite was tested on the sF (43.9%). As in sample A, the tearing strength of sample RL-A was also less affected when the tests were conducted on the sWF. In the latter case, although the damage induced by the P100 abrasive was low, the geocomposite had already been damaged by the action of *corundum*—hence the 56.8% reduction in tearing strength (which was only slightly more pronounced than that induced by the RL tests: 46.1%).

#### 3.3.2. Tests in the Cross-Machine Direction

**Figure 6:**
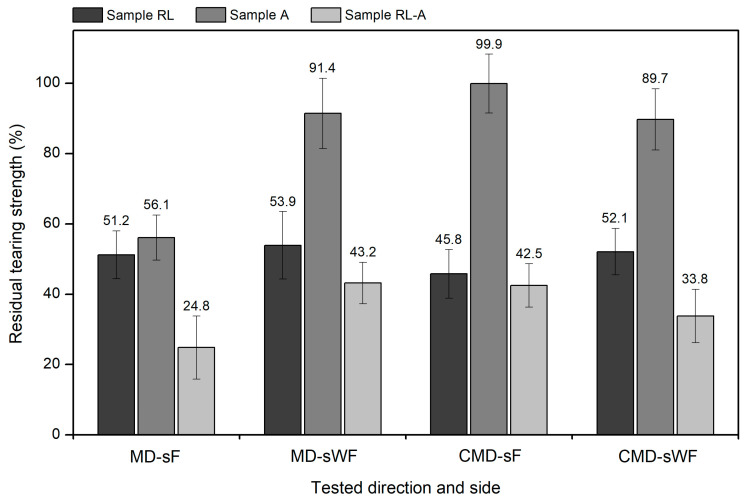
Residual tearing strength of the geocomposite.

The tearing strength of the geocomposite also underwent changes when it was tested in the CMD. In most cases, the changes were not very different from those observed when the tests were performed in the MD. Still, in some cases, there were relevant differences, which will be discussed below. To aid this discussion, Figure 6 compares, for the different conditions, the residual tensile strength of the geocomposite after the degradation tests.

For both the sF and sWF, the reduction in tearing strength promoted by the RL tests was very close when the geocomposite was tested in the MD and CMD. In fact, in all cases, the reduction in tearing strength was around 50%. This, in addition to showing that the degradation promoted by *corundum* was, for the geosynthetic under study, relatively independent of the tested direction, indicates, once again, that changing the side of the geocomposite facing the load application mechanism did not have a preponderant influence on the deterioration of its tearing behaviour.

Focusing on the results obtained in the CMD, for sample A, the tearing strength was not extremely affected when the geocomposite was tested on the sF and sWF. In the first case (sF), the MD filaments were in direct contact with the P100 abrasive, protecting the CMD filaments and geotextile (the elements responsible for tearing strength in the CMD) from degradation. Therefore, there was no reduction in tearing strength (residual tearing strength of 99.9%). In the other case (sWF), the geotextile was the element in contact with the P100 abrasive (protecting the MD and CMD filaments), resulting in a 10.3% reduction in tearing strength—a reduction similar to that observed when the geocomposite was tested in the MD (8.6%). Although, for the sWF, the change in the tested direction did not result in a significant difference in the reduction in tearing strength (promoted by the abrasive actions), the same did not occur when the geocomposite was tested on the sF. Indeed, in the latter case, the 43.9% reduction in tearing strength found in the MD contrasts with the non-variation of this property in the CMD. The reasons for this difference have already been debated: when tests were performed on the sF, the MD filaments were the elements directly exposed to the P100 abrasive, regardless of the direction under test.

Finally, regarding the RL-A sample (tests in the CMD), when the geocomposite was tested on the sF, the reduction observed in tearing strength (57.5%) was very close to that found in the RL sample (54.2%). The proximity between these reductions, and the fact that the tearing strength of sample A remained practically unchanged, shows that the deterioration in the tearing behaviour of sample RL-A seems to have been exclusively due to the RL tests. This was not observed when the geocomposite was tested on the sWF, since, in this case, the exposure to abrasion (sample A) led to a small reduction in tearing strength (10.3%). Accordingly, the reduction in tearing strength promoted by the double testing condition (66.2%) was more marked than that found in sample RL (47.9%). The comparison of the results obtained for the RL-A sample in the MD and CMD shows that, when the geocomposite was tested on the sF, the reduction in tearing strength was more pronounced when the direction tested was the MD. The opposite happened when the geocomposite was tested on the sWF.

### 3.4. Tearing Strength vs. Tensile Strength

**Figure 7:**
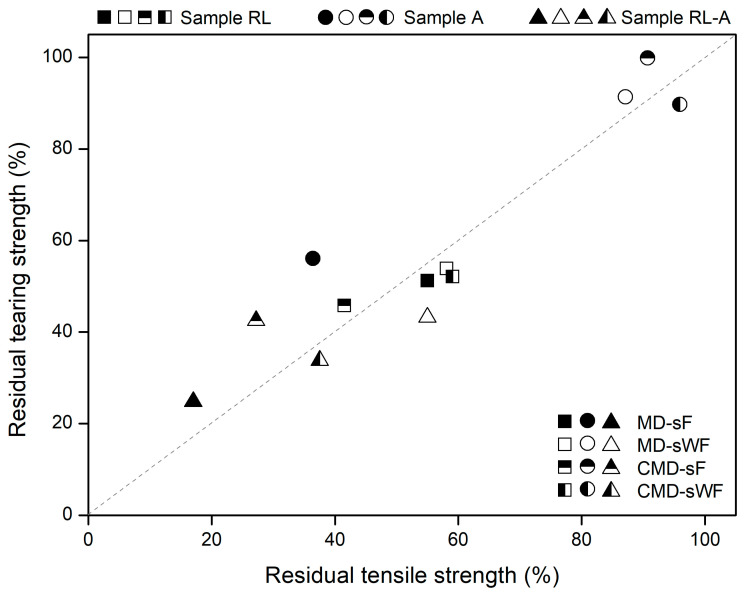
Relationship between the residual tensile and tearing strength of the geocomposite.

Having analysed the effect of the degradation tests on the tensile and tearing behaviour of the geocomposite, it becomes relevant to evaluate the existence of a relationship between the reductions found in tensile and tearing strength. Figure 7 was prepared to help accomplish this evaluation (the residual tensile strengths obtained when the geocomposite was tested in the MD were collected from Carneiro et al. [24]).

As illustrated in Figure 7, at least from a qualitative perspective, there was a relationship between the reduction in the tensile and tearing strength of the geocomposite: as one property decreased, the other also tended to decline. However, from a quantitative perspective, the relationship shown in Figure 7 is not the most rigorous. Indeed, in most cases, the percentage reductions for both properties were somewhat different (identical percentage reductions would fall on the dashed line in Figure 7). Still, in most cases (9 out of 12), the difference (in absolute value) between the residual tensile and tearing strengths was less than 10%. Therefore, for the geosynthetic under study, and not forgetting the limitations of the relationship, the reduction in tensile strength (promoted by the degradation conditions) can function as an indicator of the expected reduction in tearing strength (and vice versa).

Figure 7 also allows quick identification of the degradation conditions that had the greatest impact on the tensile and tearing strength of the geocomposite. The abrasion tests (circular symbols) tended to be the least damaging, contrasting with the double testing condition (triangular symbols), which tended to fall at the other extreme. The RL tests (square symbols) had an intermediate effect and were those where the dispersion of results was smaller—in all cases, reductions in tensile and tearing strength between ≈40% and 60%.

### 3.5. Reduction Factors

**Figure 8:**
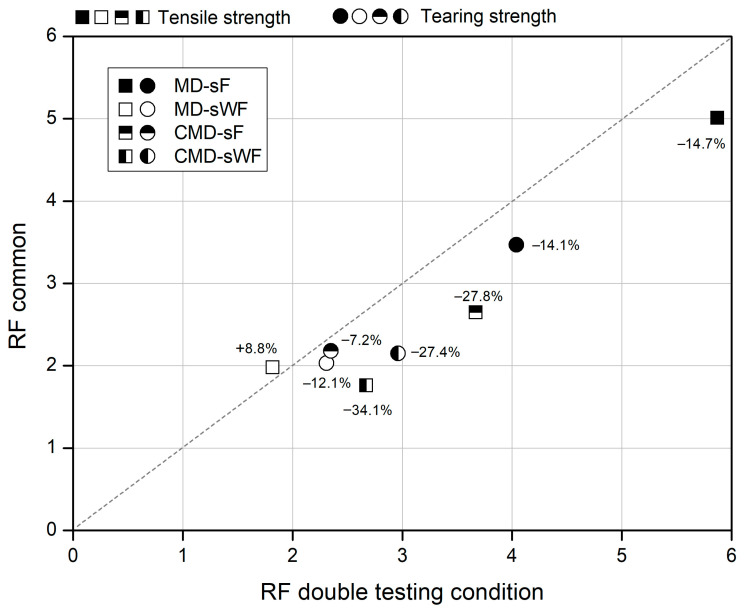
Reduction factors obtained for the combined effect of the RL and abrasion tests: common method vs. double testing condition.

The changes induced by the degradation tests in the tensile and tearing strength of the geocomposite were used to determine reduction factors, which are displayed in Table 6. For the combined effect of the RL and abrasion tests, the reduction factors were attained by using two different methods: (1) directly from the results obtained for sample RL-A (Table 6) and (2) by the common method, in which the reduction factors obtained for samples RL and A (values included in Table 6) were multiplied. The comparison of the reduction factors obtained by using both methods is illustrated in Figure 8. To broaden the comparison, the reduction factors obtained for tensile strength when the geocomposite was tested in the MD (values collected from Carneiro et al. [24]) were also included in Figure 8.

The data exhibited in Figure 8 shows that, in most cases (7 out of 8), the reduction factors (for the combined effect of the RL and abrasion tests) calculated through the common method were lower than those resulting from the double testing condition (sample RL-A). It should be noted that the percentage variations shown in Figure 8 correspond to the difference between the reduction factors obtained by using the common method relative to those found directly from the double testing condition. The smaller reduction factors associated with the common method indicate that this method may not be the most accurate solution to correctly take into account the combined effect of the RL and abrasion tests on the tensile and tearing strength of the geocomposite, with a considerable underestimation (around 30% lower) in some cases.

As shown in previous works [11,12,13], the common method may not be able to account for interactions (synergisms) between different degradation agents. If interactions are not properly quantified and underestimated reduction factors are used, the occurrence of incorrect designs is likely, which could be a source of future problems in the performance of the structures where geosynthetics are applied. Ultimately, this may lead to a reduction in the service life of those structures.

To conclude, it should be noted that the reduction factors presented in this work are exclusive to the tested geocomposite and resulted from standard laboratory degradation tests that may not accurately simulate real-world conditions. Experience has shown that there is no universal relationship between the degradation of geosynthetics in the laboratory (which occurs under controlled and reproducible conditions) and their degradation in the field, which can vary significantly from application to application. Therefore, the reduction factors reported here should not be used directly in the design (their calculation aimed to compare methods for determining reduction factors). The reduction factors to be used in the design must be determined case by case, always taking into account the specific conditions of each project and ensuring that they faithfully represent the degradation that geosynthetics will experience over time, not neglecting possible interactions between degradation agents.

## 4. Conclusions

This work assessed the effect caused by different degradation conditions (RL tests, abrasion tests and a double testing condition consisting of RL tests followed by abrasion tests) on the mechanical behaviour (tensile and tearing properties) of a reinforcement geocomposite. The influence of the tested direction and the tested side of the geocomposite was evaluated. The main findings of the work include the following:

- The degradation tests resulted in visible damage to the geocomposite (regardless of the direction or side tested), readily indicating that the reinforcement function was, in most cases, affected. The damage (type and severity) varied from test to test, anticipating different changes in the mechanical performance of the geocomposite.
- The degradation tests induced, in most cases, a deterioration in the tensile and tearing behaviour of the geocomposite.
- Regarding single tests, the reduction in tensile and tearing strength tended to be more relevant after RL tests than abrasion. The degradation induced to the geocomposite by the RL tests was relatively close, regardless of the direction or side tested.
- The double testing condition proved to be the most adverse scenario for the geocomposite, leading to the most considerable reductions in tensile and tearing strength.
- The deterioration of the tensile and tearing strength of the geocomposite depended, in some cases, on the direction tested. This was noticeable in degradation conditions that included exposure to abrasive actions.
- The tested side also influenced the degradation experienced by the geocomposite in abrasion tests. The differences were related to the structure of the geocomposite.
- An acceptable relationship was found between the decline in the tensile and tearing strength of the geocomposite. In most cases, the difference between the residual values of these two properties did not exceed 10% (in absolute values).
- With regard to reduction factors for tensile and tearing strength, the common method was, in most cases, not able to adequately quantify the combined effect of the RL and abrasion tests—the reduction factors calculated by using this method tended to be lower than those resulting from the double testing condition. The underestimation of the combined effect of degradation agents, ignoring possible interactions (synergisms) between them, may lead to incorrect designs.

The mechanical performance of the geocomposite, and thus its reinforcement capacity, was affected by most degradation tests. Knowing that its structure played a relevant role in its resistance to degradation, geocomposites with different structures will certainly have different survivability to damaging actions. To ensure that geosynthetics have adequate properties to correctly perform their functions, both in the short and long term, all types of degradation that these materials may experience over time (and the consequent effect on their properties) must be properly evaluated and taken into account during the design phase. In addition, construction procedures defined by designers and manufacturers must be duly followed to avoid or minimize degradation.

## Figures and Tables

**Table 1:** Work plan: intact and damaged samples.

Sample	Number of Specimens Tested			
	MD-sF	MD-sWF	CMD-sF	CMD-sWF
Intact	10	10	15	15
RL	10	10	15	15
A	10	10	15	15
RL-A	10	10	15	15

**Table 2:** Visual analysis of the geocomposite after the degradation tests (samples tested in the CMD).

Sample	Side	Visual Remarks
RL	sF	MD and CMD filaments: cuts; structural modifications (pulled filaments)
		Geotextile: cuts in fibres; punctures; holes
		Seams: mostly undamaged
	sWF	MD and CMD filaments: cuts; structural modifications (pulled filaments)
		Geotextile: cuts in fibres; punctures; holes
		Seams: mostly undamaged
A	sF	MD filaments: detached and cut; clusters of damaged filaments
		CMD filaments: mostly undamaged
		Geotextile: mostly undamaged
		Seams: mostly broken
	sWF	MD and CMD filaments: mostly undamaged
		Geotextile: fibres pulled and cut; texture change
		Seams: mostly undamaged
RL-A	sF	MD filaments: detached and cut; large clusters of damaged filaments
		CMD filaments: cuts, structural modifications (pulled filaments, loose filaments)
		Geotextile: cuts in fibres, punctures, holes
		Seams: mostly broken
	sWF	MD and CMD filaments: cuts; structural modifications (pulled filaments)
		Geotextile: punctures, holes, fibres pulled and cut/detached, clusters of fibres
		Seams: mostly undamaged

**Table 3:** Tensile properties of the geocomposite in the CMD.

Sample	Side with Filaments		Side without Filaments	
	*T* (kN·m^−1^)	*Ɛ_T_* (%)	*T* (kN·m^−1^)	*Ɛ_T_* (%)
Intact	43.10 ± 1.64	9.4 ± 2.0	43.10 ± 1.64	9.4 ± 2.0
RL	17.87 ± 2.11	6.6 ± 2.1	25.46 ± 1.28	7.9 ± 1.2
A	39.08 ± 1.82	7.7 ± 2.7	41.33 ± 1.96	8.3 ± 2.0
RL-A	11.74 ± 2.55	6.2 ± 2.7	16.15 ± 1.49	7.8 ± 2.4

**Table 4:** Tensile properties of the PP geotextile in the CMD (after failure of PET filaments).

Sample	Side with Filaments		Side without Filaments	
	*F* (kN·m^−1^)	*Ɛ_F_* (%)	*F* (kN·m^−1^)	*Ɛ_F_* (%)
Intact	15.65 ± 0.79	87.6 ± 13.0	15.65 ± 0.79	87.6 ± 13.0
RL	9.14 ± 1.18	49.0 ± 9.4	10.20 ± 1.19	48.8 ± 5.7
A	14.53 ± 1.04	77.8 ± 6.8	13.07 ± 1.22	90.0 ± 12.8
RL-A	8.42 ± 0.88	45.9 ± 12.0	3.61 ± 0.98	61.0 ± 20.0

**Table 5:** Tearing strength of the geocomposite.

Sample	*F_T_* (N)			
	MD-sF	MD-sWF	CMD-sF	CMD-sWF
Intact	965 ± 80	965 ± 80	871 ± 57	871 ± 57
RL	494 ± 52	520 ± 82	399 ± 54	454 ± 49
A	541 ± 42	882 ± 62	870 ± 45	781 ± 57
RL-A	239 ± 84	417 ± 46	370 ± 49	294 ± 64

**Table 6:** Reduction factors obtained for the tensile and tearing strength of the geocomposite.

Side	Sample	RF for *T*	RF for *F_T_*	
		CMD	MD	CMD
sF	RL	2.41	1.95	2.18
	A	1.10	1.78	1.00
	RL-A	3.67	4.04	2.35
sWF	RL	1.69	1.86	1.92
	A	1.04	1.09	1.12
	RL-A	2.67	2.31	2.96

## Data Availability

The data presented in this study are available on request from the corresponding author.

## Abbreviations and Notation

A—abrasion; CMD—cross-machine direction; CMD-sF—cross-machine direction and side with filaments; CMD-sWF—cross-machine direction and side without filaments; *F*—maximum tensile force (in kN·m^−1^); *F_T_*—tearing strength (in N); MD—machine direction; MD-sF—machine direction and side with filaments; MD-sWF—machine direction and side without filaments; *O*_90_—characteristic opening size (in µm); PET—polyethylene terephthalate; PP—polypropylene; RF—reduction factor; RL—mechanical damage under repeated loading; RL-A—mechanical damage under repeated loading followed by abrasion; sF—side with filaments; sWF—side without filaments; *T*—tensile strength (in kN·m^−1^); *Ɛ_D_*—elongation from which the tensile test continued only on the geotextile: damaged sample (in %); *Ɛ_F_*—elongation at maximum tensile force (in %); *Ɛ_I_*—elongation from which the tensile test continued only on the geotextile: intact sample (in %); *Ɛ_T_*—elongation at tensile strength (in %).
